# Gender effect of glucose, insulin/glucagon ratio, lipids, and nitrogen-metabolites on serum HGF and EGF levels in patients with diabetes type 2

**DOI:** 10.3389/fmolb.2024.1362305

**Published:** 2024-04-09

**Authors:** Martha Lucinda Contreras-Zentella, Martha Gabriela Alatriste-Contreras, Juan Antonio Suárez-Cuenca, Rolando Hernández-Muñoz

**Affiliations:** ^1^ Departamento de Biología Celular, Instituto de Fisiología Celular, Universidad Nacional Autónoma de México (UNAM), Mexico City, Mexico; ^2^ Departamento de Métodos Cuantitativos, División de Estudios Profesionales, Facultad de Economía, Universidad Nacional Autónoma de México (UNAM), Mexico City, Mexico; ^3^ Departamento de Medicina Interna, Hospital General “Xoco”, Secretaría de Salud (SS), Mexico City, Mexico

**Keywords:** glucose, insulin resistance, HOMA, lipids, insulin/glucagon ratio, glycosylated hemoglobin

## Abstract

Hepatocyte growth factor (HGF) exhibits potent growth-inducing properties across various tissues, while epidermal growth factor (EGF) acts as a molecular integration point for diverse stimuli. HGF plays a crucial role in hepatic metabolism, tissue repair, and offers protective effects on epithelial and non-epithelial organs, in addition to its involvement in reducing apoptosis and inflammation, underscoring its anti-inflammatory capabilities. The HGF-Met system is instrumental in hepatic metabolism and enhancing insulin sensitivity in animal diabetes models. Similarly, the EGF and its receptor tyrosine kinase family (EGFR) are critical in regulating cell growth, proliferation, migration, and differentiation in both healthy and diseased states, with EGF also contributing to insulin sensitivity. In this observational study, we aimed to identify correlations between serum levels of HGF and EGF, insulin, glucagon, glucose, and primary serum lipids in patients with type 2 diabetes mellitus (DM), taking into account the impact of gender. We noted differences in the management of glucose, insulin, and glucagon between healthy men and women, potentially due to the distinct influences of sexual hormones on the development of type 2 DM. Additionally, metabolites such as glucose, albumin, direct bilirubin, nitrites, and ammonia might influence serum levels of growth factors and hormones. In summary, our results highlight the regulatory role of insulin and glucagon in serum glucose and lipids, along with variations in HGF and EGF levels, which are affected by gender. This link is especially significant in DM, where impaired cell proliferation or repair mechanisms lead to metabolic changes. The gender-based differences in growth factors point to their involvement in the pathophysiology of the disease.

## Graphical Abstract

**Figure:**
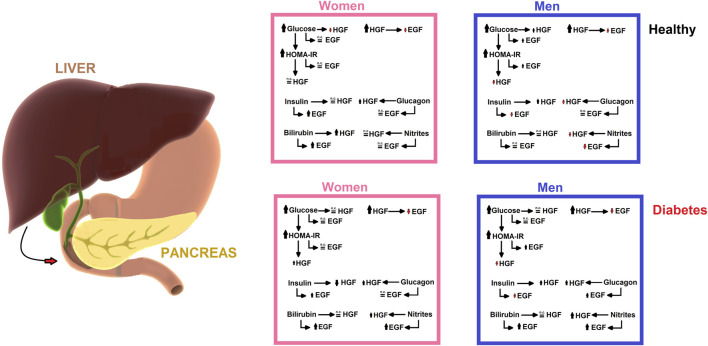
Interactions and correlations between metabolites, pancreatic hormones, and growth factors. Note the gender differences, frequently opposite among metabolites, insulin and glucagon with growth factors, as well as the effect of metabolic disturbances induced by Diabetes type 2. The size of the arrows indicates the strength of the correlation and the direction whether the correlation was straight or inverse.

## 1 Introduction

Hepatocyte growth factor (HGF) is a potent growth factor present in various tissues, including the lungs, liver, pancreas, gastrointestinal tract, brain, and kidney (Frisch et al., 2016). It is initially secreted as a single-chain precursor, pro-HGF, and its activation involves extracellular processing into a mature two-chain form (Kawaguchi and Kataoka, 2014). HGF acts as a ligand for the MET tyrosine kinase receptor (c-MET), leading to the activation of the MAPK and PI3-kinase/AKT signaling pathways (Bottaro et al., 1991; Naldini et al., 1991). This activation can occur directly through HGF binding (canonical pathway) or indirectly through interactions with other signaling cascades, such as those initiated by the epidermal growth factor receptor (EGFR) or other circulating factors (Suzuki et al., 2005).

Endogenously, HGF is essential for the self-repair of damaged visceral organs, lungs, and other tissues (Yamada et al., 2000). It also protects epithelial and non-epithelial organs, including the heart and brain, by reducing apoptosis and inflammation (Okunishi et al., 2005). These findings highlight the importance of HGF secretion balance for homeostasis and its potential therapeutic value in certain diseases, evidenced by the HGF/c-MET pathway’s clinical relevance in gastric cancer (Joo et al., 2016).

Disruption of hepatocyte c-MET signaling leads to chemotactic and inflammatory responses, underscoring HGF’s anti-inflammatory effects (Marquardt et al., 2012). Inflammation plays a significant role in type 2 diabetes mellitus (DM) pathogenesis (Kroy et al., 2014). In type 2 DM, the HGF-Met system improves insulin sensitivity in mouse models of insulin resistance, likely through direct interactions between the Met receptor and the insulin receptor, affecting hepatic glucose management (Fafalios et al., 2011). c-MET expression in β-cells increases HGF sensitivity, affecting cell growth, survival, and insulin production. HGF has both paracrine and endocrine effects, influencing β-cell homeostasis pathways (Oliveira et al., 2018). Thus, HGF is involved in β-cells' compensatory responses to conditions characterized by insulin resistance, such as obesity and pregnancy. The HGF receptor, c-Met, shares structural similarities with the insulin receptor, and the HGF signaling pathway helps regulate glucose metabolic flux in various insulin-sensitive cell types, including β-cells, enterocytes, adipocytes, hepatocytes, and myocytes, under ERK pathway regulation (Oliveira et al., 2018).

The EGF and EGFR family of receptor tyrosine kinases constitute a complex system acting as a molecular integration hub for a wide array of stimuli, including peptide ligands, metal ions, ultraviolet and gamma radiation, osmotic shock, membrane depolarization, and oxidative radicals (Siddiqui et al., 2012). Additionally, the EGFR signaling pathway plays a crucial role in regulating cell growth, proliferation, migration, and differentiation, both in normal and disease states such as cancer and diabetes-related cardiovascular dysfunction (Akhtar and Benter, 2013). In humans, the combined application of EGF and gastrin leads to the expansion and differentiation of ductal cells into Pdx-1+/insulin + cells, either through replication of existing β cells or β-cell neogenesis from ductal cells (Suarez-Pinzon et al., 2005). Furthermore, administration of EGF and gastrin to alloxan-treated mice rapidly normalized blood glucose levels within the first few days of treatment (Song et al., 2015), suggesting EGF’s role in insulin sensitivity. EGF and insulin regulate lipolysis through distinct mechanisms, as EGF (unlike insulin) increases cytoplasmic calcium signaling (Tebar et al., 1996). A potential synergistic action between HGF and EGF is observed in rodent models of liver regeneration post-partial hepatectomy, where progression from the G1 to the S phase of the cell cycle primarily involves EGFR and the HGF receptor c-Met. However, c-Met mRNA expression remains unaffected by liver fat content, while hepatic EGFR expression is significantly lower in hepatic steatosis models compared to controls (Abu Rmilah et al., 2020).

Given the hypothesis that HGF may act as a powerful inhibitor of hepatic glucose production and output in the setting of insulin resistance, thereby enhancing insulin sensitivity (Fafalios et al., 2011), our research aims to explore the potential correlation between serum HGF levels and those of insulin, glucagon, and elevated serum lipids in non-obese patients with type 2 diabetes mellitus (DM). Moreover, considering the potential interplay between HGF and EGF in activating signal transduction pathways that promote cancer cell proliferation, we are also examining possible variations in blood EGF levels among patients with type 2 DM.

## 2 Methods

### 2.1 Study population

We recruited a cohort of patients with type 2 diabetes mellitus (DM) from the outpatient clinics associated with the Ministry of Public Health (SSA, Mexico), specifically from Xoco General Hospital. The study group included 50 women and 50 men diagnosed with type 2 (non-insulin-dependent) DM. Patient selection was based on specific criteria: no active alcohol consumption, non-smoking status, and ongoing treatment with hypoglycemic agents. Additionally, we included a control group of 50 women and 50 men who were non-smokers, abstained from alcohol, and were considered healthy. Blood samples were collected from all participants after a 10-h overnight fast, during which patients did not take their morning medication. This study adhered to the ethical principles outlined in the Declaration of Helsinki (2000) by the World Medical Association and was approved by the Ethics Committee of General Hospital “Xoco” in Mexico (Ministry of Public Health), with all participants providing written informed consent.

### 2.2 Serum collection and clinical tests

Serum was separated by centrifuging uncoagulated whole blood, and the concentrations of various metabolites were quantified using standardized methods and kits provided by SPINREACT (Spain). These metabolites included glucose, cholesterol, and triacylglycerols (TG), along with the activities of enzymes indicative of liver damage, specifically alanine (ALT-GPT) and aspartate (AST-GOT) aminotransferases. Insulin concentrations were assessed with a kit from RayBiotech (United States of America), and HbA1c levels were determined using a kit from BioSys-Kovalent (Brazil) (Table 1). (Ramírez-Zamora et al., 2013; Contreras-Zentella et al., 2019).

**TABLE 1:** Clinical parameters and serum levels of HGF, EGF, insulin, and nitrogen-related compounds in control subjects and patients with Type 2 Diabetes Mellitus, with results expressed as medians. Abbreviations used: TG (triacylglycerols). Statistical analysis employed the non-parametric Kolmogorov-Smirnov (KS) test, without adjustment for multiple comparisons. Data are expressed as medians and their distributions are arranged by rows, categorizing various determinations by gender and group within serum samples.

Parameter	Control Subjects		Patients with type 2 DM	
	Women	Men	Women	Men
Age (years)	42	48	49	50
Number of subjects	50	50	50	50
Glucose (mg • dL^-1^)	88	94	149	142
HOMA - IR	1.73	1.48	3.03	2.90
Hb A_1c_ (mmoles • mole^-1^)	25	28	75	70
Cholesterol (mg • dL^-1^)	150	138	185	179
TG (mg • dL^-1^)	143	148	187	175
Albumin (g • dL^-1^)	4.3	4.9	3.4	4.2
Total Bil (mg • dL^-1^)	0.48	0.63	0.15	0.31
Direct Bil (mg • dL^-1^)	0.19	0.20	0.11	0.24
HGF (nmoles • m1^−1^)	8.7	9.2	20.3	14.1
EGF (ng • ml^-1^)	1.2	1.4	2.1	1.8
Insulin (nUnits • m1^−1^)	6.4	4.6	8.4	10.1
Glucagon (pg • mn	38.2	35.4	31.5	29.7
Insulin I Glucagon (nUnits • m1^−1^)	82.7	80.1	109.3	128.2
Nitrites (µmoles • dL^-1^)	1.3	1.9	5.6	4.4
Ammonia (µmoles • dL^-1^)	4.5	4.9	9.4	7.7

### 2.3 Glucagon, HGF, and EGF measurements

Serum glucagon levels were determined using an ELISA kit from RayBiotech. Similarly, levels of HGF and EGF were measured using ELISA kits from BioSys-Kovalent (Table 1).

### 2.4 Statistical analysis

A total of 84 determinations were conducted, with each dataset comprising 40 values sorted by metabolite, blood compartment, group, and gender. We utilized a pooling strategy, collecting blood samples from each participant in the patient and control groups, and derived determinations from clustered samples. It is crucial to recognize that the findings and conclusions are derived from cluster-level data, not individual patient data. The complete datasets are available in the Data Sets section of the Open Science Framework project (https://osf.io/a6cd3/?view_only=90dfd426c06648f5bbb96470c689d106). Statistical analysis was carried out using Python 2.7, along with the Numpy and Scipy libraries. Although it is common to presume a normal or t-Student distribution for data, justifying the use of only 30 data points, our statistical analysis did not rely on any pre-assumed probability distribution. Clinical data on metabolites often show a skewed, rather than normal, distribution (Contreras-Zentella et al., 2019). Our initial step was to determine the most suitable distribution for the data. We also refrained from presupposing linear or any specific relationship between variables due to the lack of evidence. To analyze statistical differences based on gender, we fitted the data to various continuous variable distributions using maximum likelihood estimation to derive estimators for shape, location, and scale parameters. The distributions considered for fitting included beta, exponential, exponential-Weibull, exponential-power law, Gilbrat, logistic, lognormal, normal, Pareto, power law, Weibull minimum, and Weibull maximum (Clauset et al., 2009). As a result, we used the Spearman correlation coefficient, which does not presuppose any predetermined relationship. Given our use of the Spearman correlation coefficient for examining variable associations, it was logical to apply polynomial fitting to identify the best mathematical relationship between variables, thereby providing the most accurate approximation and visualizations. Polynomial fitting describes the relationship between a dependent variable (y) and an independent variable (x) as an nth-degree polynomial (Wasserstein and Lazar, 2016):

$$y=c_{n}x^{n}+c_{n-1}x^{n-1}+\ldots +cx+c_{0}$$

Curve fitting involves constructing a polynomial curve that best matches a set of data points. This optimal fit yields a mathematical function that most accurately represents the relationship between two variables, enhancing our understanding of variable behavior. Although related, curve fitting to a polynomial differs from regression analysis, which is more concerned with statistical inference. In polynomial curve fitting, we select coefficients that provide the best match between the curve and the data, aiming to minimize the residuals between the observed data (y) and the fitted curve. The optimal curve is the one that yields the minimum residuals. We conducted polynomial fitting for polynomials of degrees 1, 2, and 3, and reported the one that showed the best performance.

Spearman correlation coefficients and their corresponding *p*-values were calculated since the Spearman correlation evaluates monotonic relationships and does not presume a linear correlation between the two datasets. This method is particularly relevant for analyzing data with type 2 diabetes mellitus (DM), as it allows for the assessment of relationships without assuming linearity (Mann and Whitney, 1947; Fay and Proschan, 2010).

## 3 Results

### 3.1 clinical parameters and serum levels of HGF, EGF, insulin and glucagon in healthy subjects

The statistical analysis of clinical parameter values indicated their probability distribution or adjustment lines. Most distributions showed a skewed pattern (Supplementary Material S1), leading to the use of the median as a comparative measure for datasets (Supplementary Material S1, 2). Compared to control subjects, the medians of all clinical parameters were within normal ranges. Notably, insulin levels were lower in men than in women, whereas total bilirubin showed the reverse trend (Table 1). This resulted in minor gender differences in the median values of these clinical parameters among healthy control subjects.

Gender-based differences in the control group were noted in the statistical analysis of distributions for glucose, HOMA-IR, HbA1c, and triacylglycerols (TG) (Supplementary Material S2). Spearman correlation coefficients indicated weak correlations between gender and HOMA-IR, TG, as well as HbA1c and insulin, with both showing negative associations, indicating opposite trends for men and women (rho coefficient = −0.236 and −0.28, respectively). Interestingly, glucagon exhibited the same distribution pattern by gender (power law) but without any correlation (rho coefficient = −0.03) (Table 1; Supplementary Material S2-4). These results suggest differences in glucose management and related molecules between healthy men and women, likely due to different hormonal patterns.

In the control group, median hepatocyte growth factor (HGF) levels were similar between genders, while epidermal growth factor (EGF) levels were slightly higher in men (18%; Table 1). The distribution of HGF among healthy subjects showed no significant gender differences or correlation (beta distribution; *p*-values of 0.550 and 0.999, respectively; Supplementary Material S1, rho coefficient = 0.0868; Supplementary Material S4). In contrast, EGF distributions differed by gender (Weibull min and expWeibull, respectively), with a weak correlation observed between genders (rho coefficient = 0.2496; Supplementary Material S2, 4).

### 3.2 Clinical parameters and serum levels of HGF, EGF, insulin, and glucagon in patients with type 2 DM

In patients with type 2 diabetes mellitus (DM), median HbA1c levels exceeded the normal range (>35 mmols/mol), reflecting high blood sugar levels. Both hyperglycemia (>120 mg/dL) and hyperinsulinemia (>8 µU/mL) were observed, with hyperinsulinemia predominantly higher in men. Glucagon levels were comparable across genders but slightly lower than those in control subjects, consistent with diabetic pathology (Table 1). Statistical analysis showed gender-specific distributions for glucose, HOMA-IR, HbA1c, and triglycerides in type 2 DM patients (Supplementary Material S2). Weak to moderate correlations between various parameters and gender were noted, with Spearman correlation coefficients ranging from 0.194 to 0.322. Specifically, HbA1c displayed a negative Spearman correlation, suggesting differing trends between diabetic men and women (Supplementary Material S4). The Spearman correlation coefficient revealed a weak negative correlation between genders in type 2 DM (rho coefficient = −0.114; Supplementary Material S2-4).

Unlike in controls, serum glucagon levels in DM patients varied between genders, with a strong positive Spearman correlation observed between diabetic men and women (rho coefficient = 0.770).

Cholesterol, albumin, and both total and direct bilirubin levels in DM patients were within normal limits, though their distributions varied by gender. There was no correlation for cholesterol, albumin, and direct bilirubin between diabetic men and women (rho coefficients ranged from 0.069 to 0.256). A moderate negative correlation was noted for total bilirubin (rho coefficient = −0.359; Supplementary Material S4).

For hepatocyte growth factor (HGF) and epidermal growth factor (EGF) in type 2 DM patients, median levels were elevated (2.2-times for HGF and 1.7-times for EGF). Moreover, distributions varied between genders (Supplementary Material S1, 2), with Spearman correlation coefficients showing a weak correlation for HGF and no significant correlation for EGF (Supplementary Material S4).

### 3.3 Serum levels of nitrogen-related metabolites (nitrites and ammonia) in patients with type 2 DM

Serum nitrite levels were similar between healthy men and women but were elevated in patients with type 2 diabetes mellitus (DM), especially in women (Table 1; Supplementary Material S1). The Spearman correlation coefficient revealed a weak correlation by gender for this nitrogen-related metabolite in both healthy individuals and those with type 2 DM (Supplementary Material S4).

Notably, serum ammonia levels were higher in patients with type 2 DM (Table 1; Supplementary Material S1), with the Spearman correlation coefficient showing a weak negative correlation under these conditions. Among healthy subjects, the Spearman correlation coefficient for gender demonstrated a strong correlation (rho coefficient = 0.667) (Supplementary Material S4).

**FIGURE 1:**
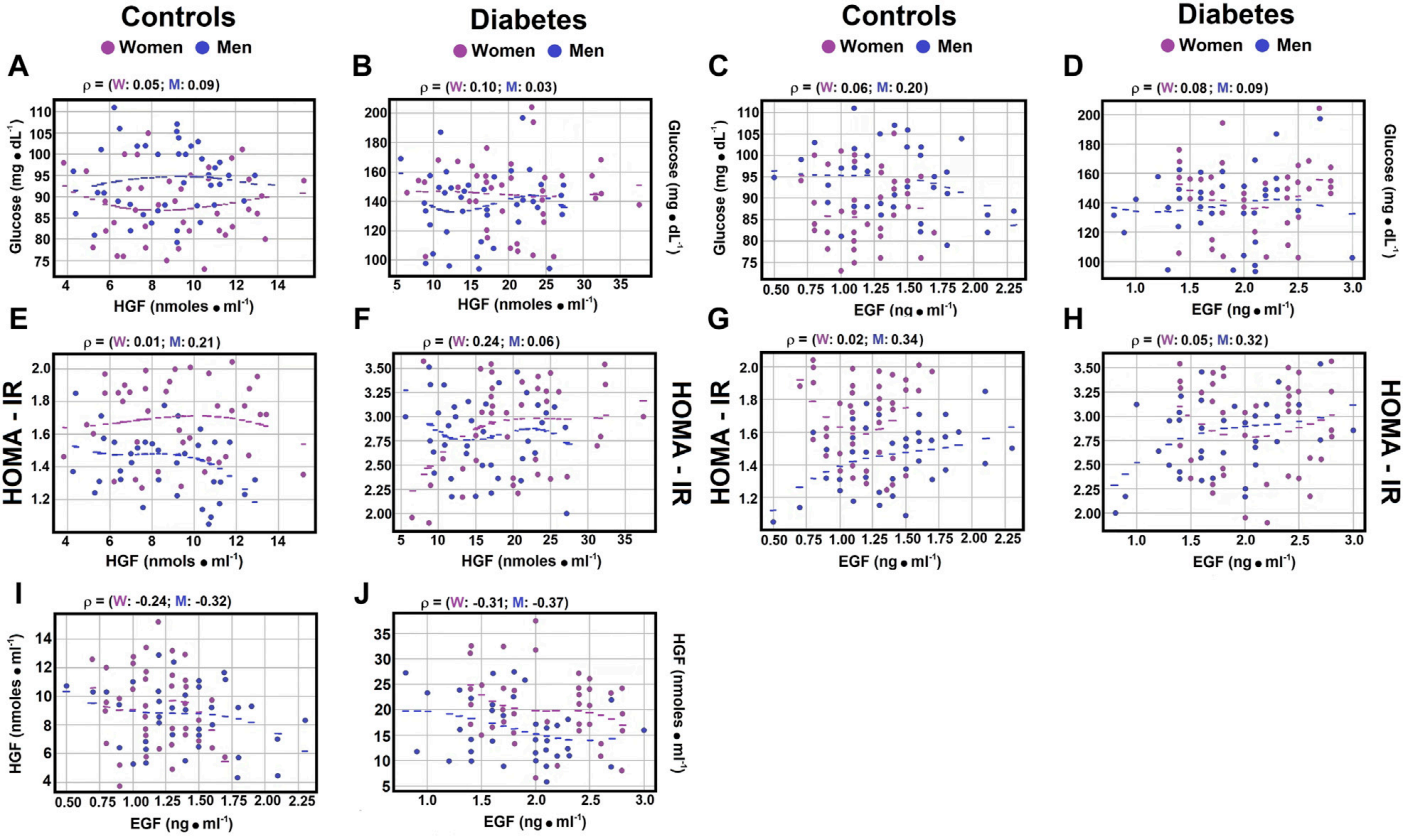
Correlations between serum glucose levels, HOMA-IR, and growth factors. The figure displays scatter plots illustrating key correlations (dots) and their optimally fitted polynomials (lines), with blue representing males and magenta representing females. In every instance, a third-degree polynomial best captured the relationship between the variables, despite a very low R-squared value. This low R-squared underscores the weak, yet nonlinear relationship previously identified through Spearman correlations; the most suitable polynomial was of degree 3. The selection criteria for the best polynomial were the lowest residuals and the highest R-squared value. The scatter plots show the Spearman correlation coefficient between serum glucose and HGF levels in control subjects Panel **(A)**, and in patients with type 2 DM Panel **(B)**, as well as the relationship between serum glucose and EGF levels in controls Panel **(C)** and diabetic patients Panel **(D)**. The correlation between the HOMA-IR index and HGF levels is shown for control subjects Panel **(E)** and patients with type 2 DM Panel **(F)**, with the correlations between HOMA-IR and EGF levels depicted for controls Panel **(G)** and diabetic patients Panel **(H)**. Notably, significant correlations were found between serum levels of HGF and EGF both in control subjects Panel **(I)** and patients with type 2 DM Panel **(J)**. Rho values for each gender in control subjects or in patients with type 2 DM at the top of each panel.

Correlations between indicators of type 2 DM and serum growth factors. As previously discussed, hepatocyte growth factor (HGF) is pivotal in the development of type 2 diabetes mellitus (DM), serving as a significant inhibitor of hepatic glucose production in individuals with insulin resistance (Nakamura et al., 1998; Fafalios et al., 2011). Similarly, epidermal growth factor (EGF), which activates the epidermal growth factor receptor (EGFR), is involved in diabetes onset, vascular dysfunction, and the pathogenesis of type 2 DM (Wild et al., 2004; Breindel et al., 2013). In exploring the potential correlation between HGF and EGF levels, weak negative correlations were found in control subjects between serum HGF and EGF, regardless of gender (Figure 1I; Supplementary Material S4; rho coefficient = −0.1364 in women, and rho coefficient = −0.179 in men). Notably, for patients with type 2 DM, the correlations were stronger for both women (rho coefficient = −0.2144) and men (rho coefficient = −0.2687) compared to control subjects (Figure 1J; Supplementary Material S4).

Moreover, the correlation between the Homeostatic Model Assessment for Insulin Resistance (HOMA-IR) and HGF levels showed a weak negative correlation in healthy men (rho coefficient = −0.2131) (Figure 1E; Supplementary Material S4), thereby reinforcing the previously obtained results and further highlighting the differences related to gender.

### 3.4 Correlations of serum glucose, insulin, glucagon, and the insulin/glucagon ratio with HGF levels

**FIGURE 2:**
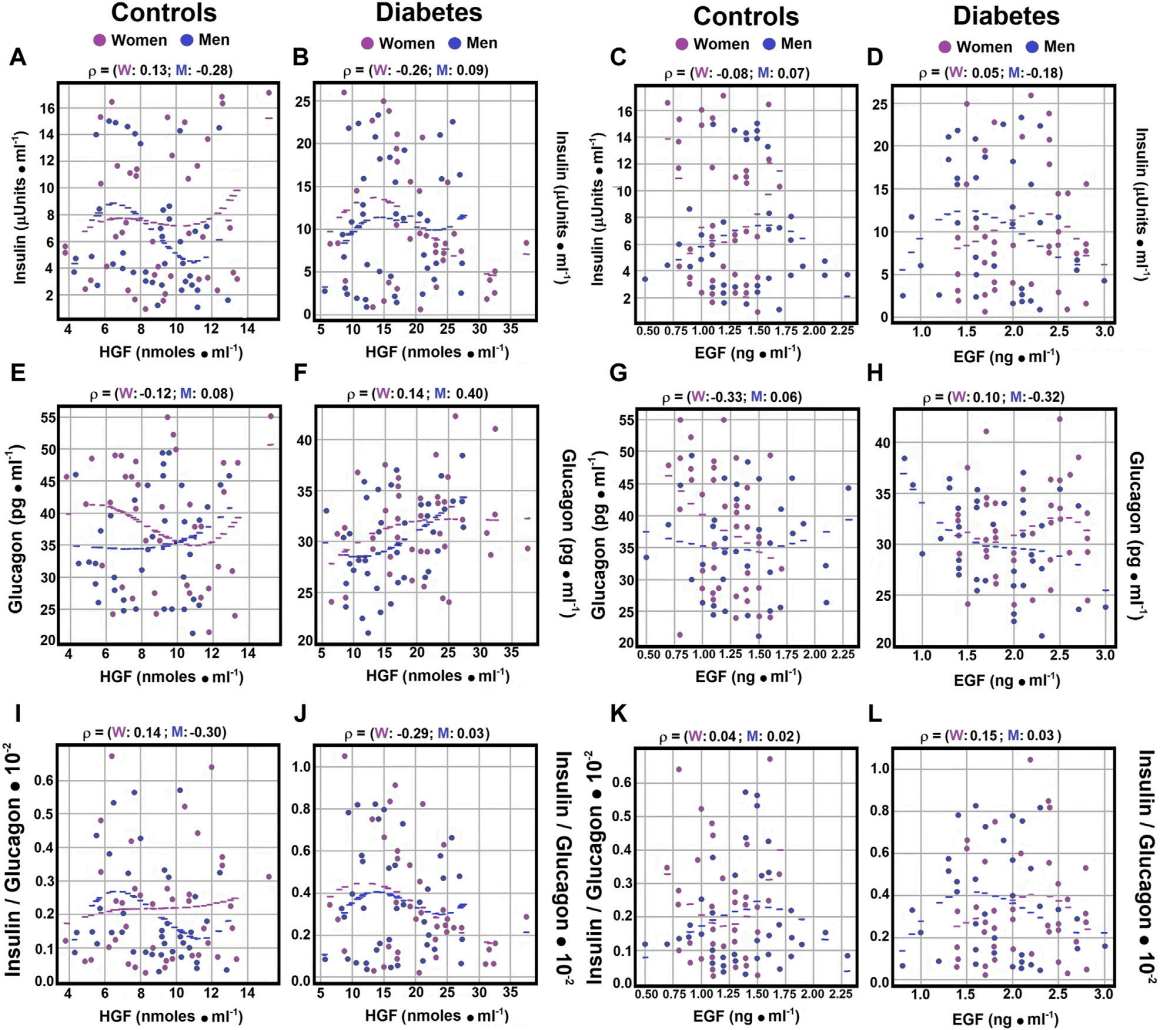
Correlations of Serum Insulin and Glucagon Levels with Growth Factors. Using the statistical analysis outlined in Figure 1, scatter plots illustrate Spearman’s correlation coefficients between serum insulin and HGF levels in both control subjects panel **(A)** and diabetic patients panel **(B)**, as well as between serum insulin and EGF levels in control subjects panel **(C)** and patients with type 2 DM panel **(D)**. Correlations between serum glucagon and HGF levels are displayed for both controls panel **(E)** and diabetic patients panel **(F)**. Panel **(G)** exhibits correlations between serum glucagon and EGF levels in controls, while panel **(H)** presents those observed in patients with type 2 DM. The insulin/glucagon ratio demonstrates correlations with serum HGF levels in both controls panel **(I)** and diabetic patients panel **(J)**. Lastly, panel **(K)** illustrates correlations of the insulin/glucagon ratio with serum EGF levels in both control subjects and patients with type 2 DM panel **(L)**. Rho values for each gender in control subjects or in patients with type 2 DM at the top of each panel.

Regarding the correlation between serum glucose levels and hepatocyte growth factor (HGF), no significant correlations were noted in healthy controls or patients with diabetes mellitus (DM), independent of gender (Figures 1A,B; Supplementary Material S4). The Spearman correlation coefficients for control subjects showed a negligible to weak, yet opposite, correlation between glucagon and HGF (rho coefficients 0.0773 for men and −0.1225 for women). In women with type 2 DM, a weak correlation was observed between glucagon and HGF (rho coefficient 0.1441; Figure 2F; Supplementary Material S4), while men with DM exhibited a moderate correlation between these variables (rho coefficient 0.3981; Supplementary Material S4). These findings indicate possible gender-specific differences in the interaction between glucagon and HGF among individuals with DM compared to controls.

As for the correlation between insulin and HGF, Spearman correlation coefficients showed a negligible to weak correlation for men with DM and control women (Figures 2A,B; Supplementary Material S4). Conversely, healthy men and women with diabetes demonstrated weak and negative correlations (rho coefficients of −0.2777 and −0.2639, respectively) between insulin and HGF (Figures 2A,B; Supplementary Material S4). This underscores distinct interactions between insulin and HGF based on gender (Table 1), highlighting its importance in the context of type 2 DM.

In analyzing the Spearman correlation coefficient between HGF and the insulin/glucagon ratio, weak negative correlations were identified in control men (rho coefficient of −0.2963; Supplement 4), whereas control women exhibited weak positive correlations (rho coefficient of 0.1396; Figure 2I; Supplementary Material S4). The correlation patterns among DM patients varied significantly by gender, showing no correlation for men and a weak negative correlation for women (rho coefficients of 0.0254 for men and −0.2897 for women; Figure 2J; Supplementary Material S4). These outcomes further confirm gender-specific variations noted in earlier analyses, emphasizing insulin’s key role in the interplay with HGF.

### 3.5 Correlations of EGF with Serum glucose, insulin, glucagon, and the insulin/glucagon ratio

Regarding the correlation between serum glucose levels and epidermal growth factor (EGF), weak correlations were noted in both healthy controls and patients with diabetes mellitus (DM), with no significant differences by gender (rho coefficients ranging from 0.073 to 0.2553) (Figures 1C,D; Supplementary Material S4). Analysis of the correlation between the Homeostatic Model Assessment for Insulin Resistance (HOMA-IR) and EGF levels showed no significant correlation in control and diabetic women (rho coefficients of 0.0247 and −0.0516, respectively; Figures 1G,H; Supplementary Material S4). However, both control men and diabetic male patients displayed moderate Spearman correlation coefficients with HOMA-IR and EGF (rho coefficients of 0.3358 and 0.3226, respectively; Figures 1G,H; Supplementary Material S4).

No significant Spearman correlation coefficients were found between EGF and insulin levels in control men and diabetic women (rho coefficients of 0.0651, −0.0837, and 0.0498, respectively; Figures 2C,D; Supplementary Material S4). A weak and negative Spearman correlation coefficient was observed between EGF and insulin levels in diabetic individuals (rho coefficient of −0.1835; Figure 2D; Supplementary Material S4). Moderate and negative Spearman correlation coefficients were identified between EGF and glucagon levels in healthy women and diabetic men (rho coefficients of −0.3316 and −0.3182, respectively; Figures 2G,H; Supplementary Material S4). In contrast, healthy men and diabetic women showed no significant correlation (rho coefficients of 0.0606 and 0.0991, respectively; Supplementary Material S4).

**FIGURE 3:**
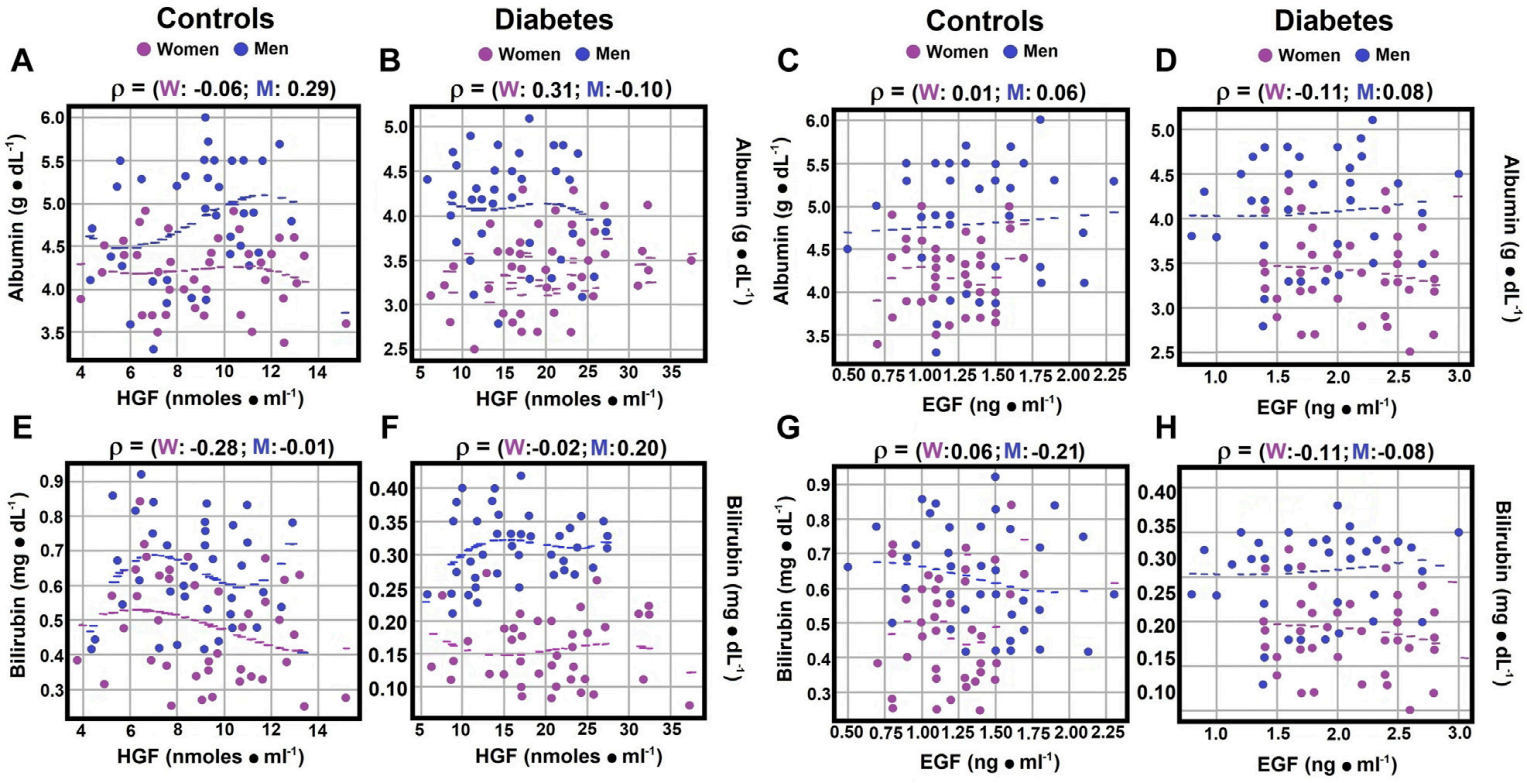
Correlations between serum albumin and bilirubin (direct) levels and growth factors. Using the statistical analysis outlined in Figure 1, the scatter plots illustrate Spearman’s correlation coefficients for serum albumin and HGF levels in both the control group panel **(A)** and patients with type 2 diabetes panel **(B)**. Correlations between serum albumin and EGF levels are depicted in control subjects panel **(C)** and diabetic patients panel **(D)**. Some correlations between total bilirubin and HGF levels were observed in both controls panel **(E)** and patients with type 2 diabetes panel **(F)**. Correlations between total bilirubin and EGF levels were also identified in control subjects panel **(G)** and diabetic patients panel **(H)**. Rho values for each gender in control subjects or in patients with type 2 DM at the top of each panel.

Furthermore, negligible to weak Spearman correlation coefficients were detected between EGF levels and the insulin/glucagon ratio across all groups, with a negative correlation observed specifically in diabetic men (rho coefficient of −0.1456) (Figure 3K-L; Supplementary Material S4). These results indicate the existence of correlations between the growth factors HGF and EGF and hormones related to metabolic disorders, highlighting the complex interactions within metabolic regulation.

### 3.6 Correlations of serum albumin, bilirubin (total), ammonium and nitrites levels with HGF

Serum albumin and hepatocyte growth factor (HGF) levels demonstrated no significant correlation for control women and diabetic men (rho coefficients of −0.0580 and −0.0995; Figures 3A,B; Supplementary Material S4). However, a weak Spearman correlation coefficient was noted between serum albumin and HGF for control men (rho coefficient 0.2931), and a moderate correlation was observed for diabetic women (rho coefficient 0.3085; Figures 3A,B; Supplementary Material S4). Total bilirubin and HGF levels showed no correlation for control men and a moderate correlation for control women (rho coefficients of −0.006 for men, and −0.277 for women), while a weak correlation was identified for both genders in diabetic patients, with a negative correlation for women (−0.1128) and a positive one for diabetic men (0.2101; Figures 3E,F; Supplementary Material S4).

**FIGURE 4:**
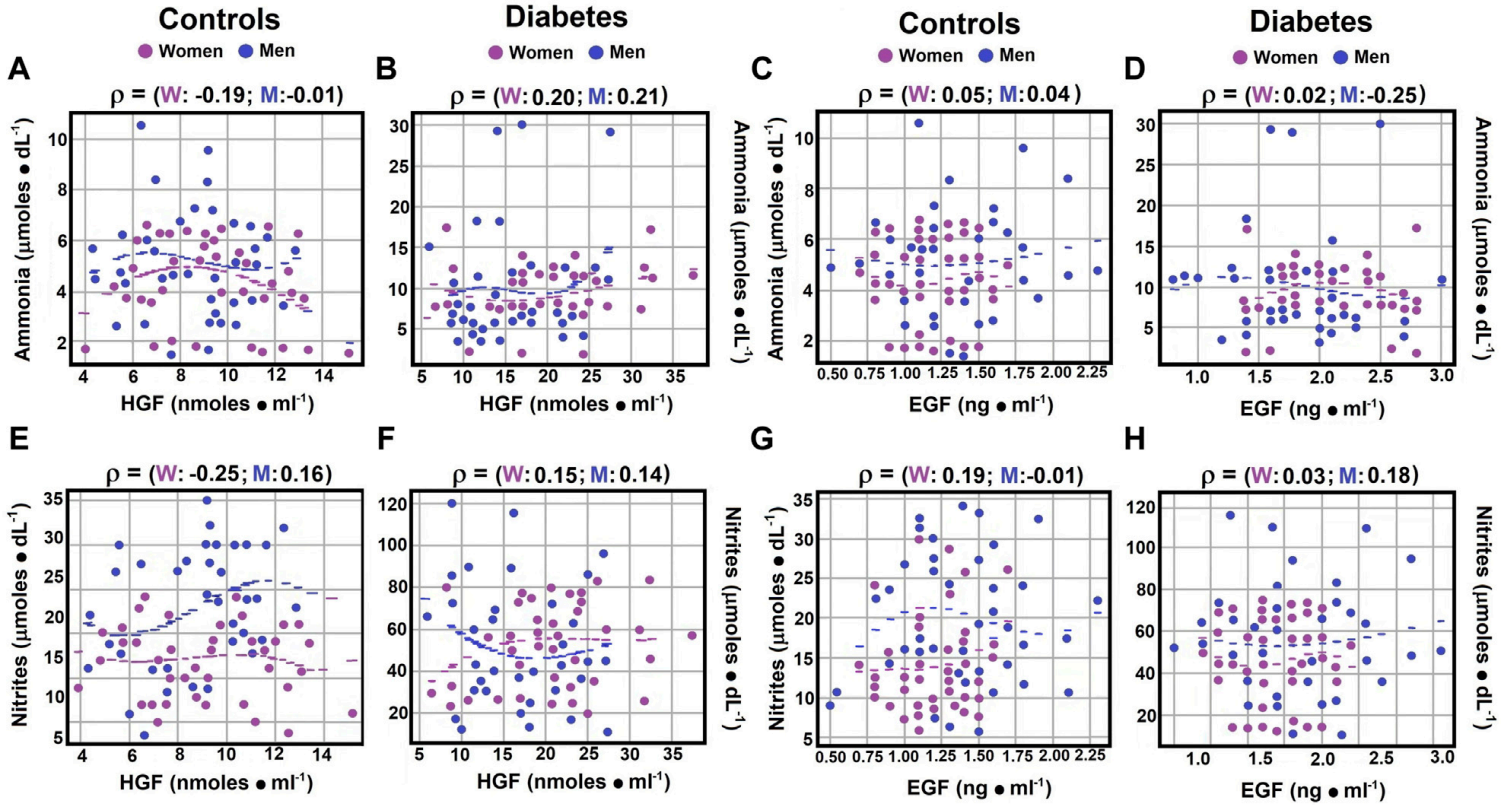
Correlations between serum ammonia and nitrite levels and growth factors. Using the statistical analysis outlined in Figure 1, the scatter plots illustrate Spearman’s correlation coefficients for serum ammonia levels compared to HGF levels in both controls panel **(A)** and diabetic patients panel **(B)**. Correlations between serum ammonia levels and EGF levels are depicted for control subjects in panel **(C)** and for diabetic patients in panel **(D)**. Regarding serum nitrite levels *versus* HGF levels, correlations are displayed for both control subjects panel **(E)** and patients with type 2 diabetes panel **(F)**. Similarly, correlations between nitrite levels and EGF levels are shown for control subjects in panel **(G)** and for diabetic patients in panel **(H)**. Rho values for each gender in control subjects or in patients with type 2 DM at the top of each panel.

Regarding the correlation between ammonia levels and HGF, control men showed no correlation (rho coefficient −0.0016), whereas control women and diabetic men exhibited weak negative correlations (rho coefficients of −0.1865 and −0.1396, respectively). Diabetic women displayed a weak but positive Spearman correlation coefficient (rho coefficients of 0.2009 and 0.2022, respectively; Figures 4A,B; Supplementary Material S4).

In terms of nitrites, weak correlations with HGF were observed across all groups (rho coefficients ranging from 0.1396 to 0.2534; Supplementary Material S4), with negative correlations noted for diabetic men and control women (Figures 4E,F; Supplementary Material S4), suggesting nuanced interactions between these biochemical markers and HGF across different health and disease states.

### 3.7 Correlations between serum albumin, direct bilirubin, ammonium, nitrites levels, and EGF

No significant correlation was found between serum albumin and epidermal growth factor (EGF) levels, with rho coefficients ranging from 0.0057 to 0.0827 (Supplementary Material S4). However, diabetic women showed a weak negative correlation (rho coefficient of −0.111; Figures 3C,D; Supplementary Material S4). For serum total bilirubin and EGF levels, diabetic men exhibited no correlation, while a weak negative correlation was noted in other experimental groups, with rho coefficients ranging from −0.2120 to −0.0856 (Figures 3G, H; Supplementary Material S4).

Regarding the correlation between serum ammonia and EGF levels, a null correlation was observed in both genders and in diabetic women. Diabetic men, on the other hand, showed a weak negative correlation (rho coefficient of −0.2528; Figures 4C,D; Supplementary Material S4). Concerning the correlation between nitrites and EGF levels, both diabetic men and control women demonstrated weak positive correlations, with Spearman correlation coefficients of 0.1779 and 0.1851, respectively (Figures 4G,H; Supplementary Material S4).

### 3.8 Correlations between serum cholesterol, TG, and growth factors

**FIGURE 5:**
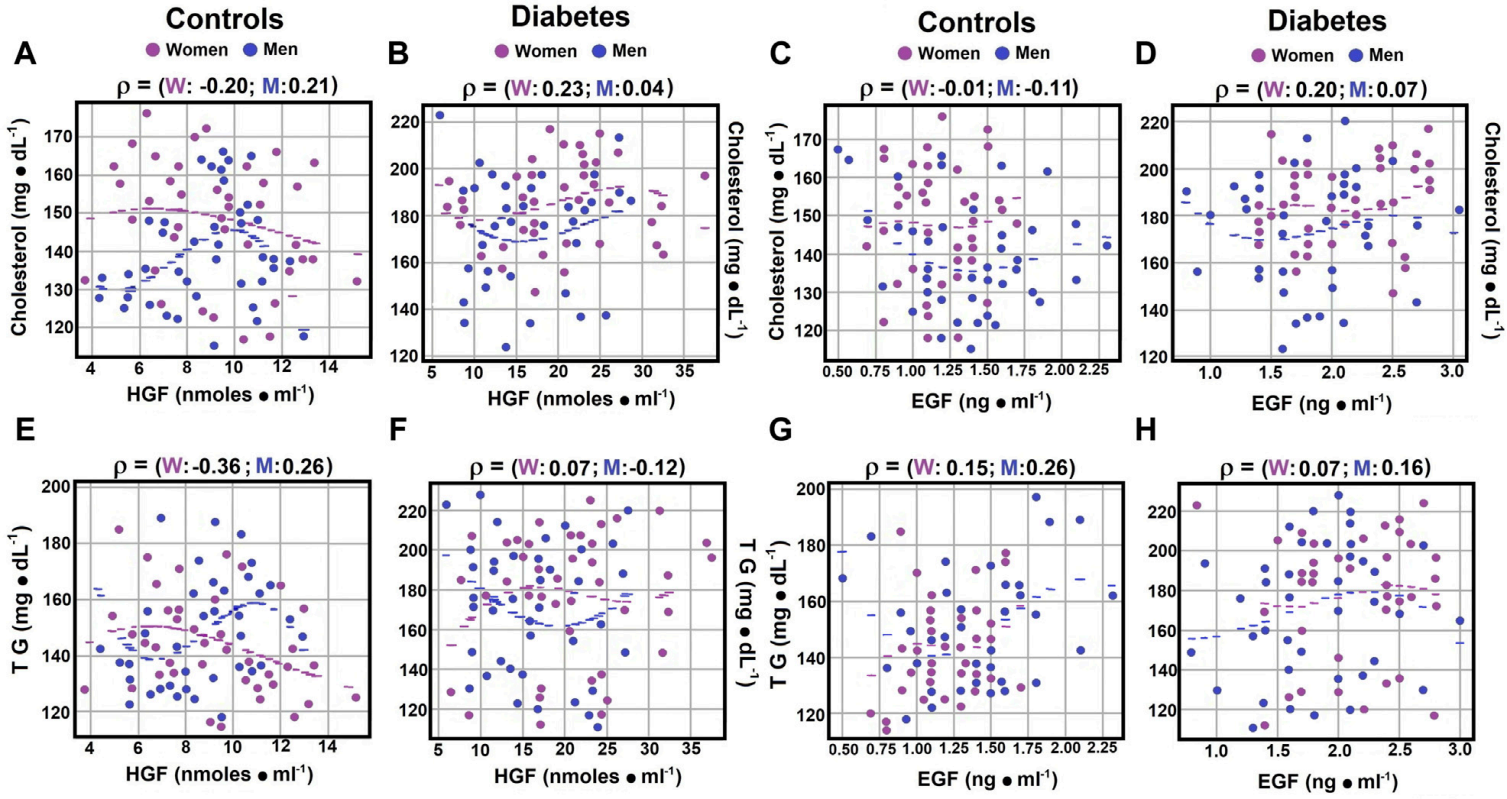
Correlations between serum cholesterol, TG, and growth factors. Using the statistical analysis described in Figure 1, the scatter plots illustrate Spearman’s correlation coefficients for serum cholesterol levels in both control subjects panel **(A)** and patients with type 2 diabetes mellitus panel **(B)**. When investigating cholesterol levels in relation to EGF levels, no significant correlation was observed in either control subjects or diabetic patients panels **(C, D)**. Concerning serum triglyceride (TG) levels in relation to HGF levels, correlations in control subjects are presented in panel **(E)**, while those in diabetic patients are displayed in panel **(F)**. However, when correlating TG levels with EGF levels, no significant correlations were found in either group panels **(G, H)**. Rho values for each gender in control subjects or in patients with type 2 DM at the top of each panel.

Correlations between serum lipids and growth factors were explored. No significant correlation was found between serum total cholesterol and hepatocyte growth factor (HGF) levels in diabetic men (rho coefficient of 0.0428; Figure 5B; Supplementary Material S4). Weak negative correlations were noted in control women, whereas control men and diabetic women exhibited weak but positive correlations (rho coefficients of −0.1965, 0.2119, and 0.2339, respectively; Figures 5A,B; Supplementary Material S4).

In contrast to cholesterol, triglycerides (TG) showed different correlations with HGF levels. Control women had a moderate negative correlation (rho coefficient of 0.3252), diabetic women showed no significant correlation (rho coefficient of 0.0660), and among men, weak correlations were found with TG and HGF, negative for diabetic men (rho coefficients of −0.1178 and 0.2632; Figures 5E,F; Supplementary Material S4).

Regarding the correlation between serum cholesterol and epidermal growth factor (EGF) levels, control women and diabetic men exhibited no significant correlations (rho coefficients of −0.0023 and 0.0664, respectively; Supplementary Material S4). Control men and diabetic women showed weak correlations, but in opposite directions (rho coefficients of −0.1107 and 0.1988, respectively; Figures 5C,D; Supplementary Material S4). Serum TG levels showed no significant correlation with EGF in diabetic women (rho coefficient of 0.0737), while in other groups, weak correlations were observed (rho coefficients ranging from 0.1491 to 0.2553; Figures 5G,H; Supplementary Material S4). These results suggest that HGF levels may have a more significant role in the regulation of serum triglyceride levels.

## 4 Discussion

In this study, we observed gender differences in the management of glucose, albumin, nitrogen-related molecules, and hormones such as insulin and glucagon. Notably, serum insulin levels were lower in men than in healthy women, whereas total bilirubin levels were higher in men. These disparities could be linked to the influence of sexual hormones.

In the context of Diabetes mellitus, traditional therapies often achieve limited success due to the inactivation of β-cells within the pancreatic islets, leading to a range of associated complications and systemic disorders (Fioretto et al., 1998; Mellado-Gil et al., 2011). However, it has been proposed that the hepatocyte growth factor (HGF)–Met axis functions similarly to the insulin–INSR system, especially in regulating hepatic glucose metabolism, suggesting a direct interaction between Met and INSR. Supporting this theory, administering HGF to mice has demonstrated modulation of hepatic mRNA expression of enzymes involved in glucose and lipid metabolism, thereby mimicking the effects of insulin (Fafalios et al., 2011). Moreover, the HGF-Met signaling pathway indirectly supports glucose homeostasis by enhancing β-cell proliferation and functionality (Zhang et al., 2015). Thus, evaluating the effectiveness and practicality of HGF in the clinical management of Diabetes mellitus is essential (Fafalios et al., 2011).

Our results show varying associations between serum glucose levels and HGF in the control group, with men exhibiting a weak correlation and women displaying a moderate, yet opposite, correlation. Examining the relationship between the Homeostatic Model Assessment for Insulin Resistance (HOMA-IR) and HGF in the control group revealed no correlation in control women, possibly indicating a reduced correlation between insulin and HGF in these subjects. Among diabetic patients, gender differences were again apparent. Diabetic men showed a weak negative correlation between glucose levels and HGF, while in women, this correlation was non-existent. Conversely, the relationship between HOMA-IR and HGF showed no correlation in diabetic men but a weak correlation in diabetic women.

In relation to epidermal growth factor (EGF), weak correlations were noted between glucose levels and the growth factor in both genders of control subjects and in men with type 2 diabetes mellitus (DM); no correlation was observed for diabetic women (Figure 1). Furthermore, when correlating the Homeostatic Model Assessment for Insulin Resistance (HOMA-IR) with EGF, gender-dependent differences emerged in both healthy controls and diabetic patients (Figure 1). Male subjects, both control and diabetic, showed an increased relationship with EGF up to a moderate correlation, while female subjects, both control and diabetic, exhibited a loss of correlation. These findings highlight significant gender-mediated differences in the signaling actions of the studied growth factors.

Crosstalk between the MET and EGFR pathways, involving cell survival, proliferation, and migration, has been implicated in the development and progression of cancer in certain tissues (Zhang et al., 2009). In the context of DM, EGF has been shown to significantly promote the expansion of pancreatic progenitors, resulting in a threefold increase in the number of PDX1-positive cells (Otonkoski et al., 1996). HGF treatment has also been demonstrated to enhance insulin production by differentiated β-cells (Jiang et al., 2007). Moreover, the combined action of HGF and EGF encourages β-cell differentiation during pancreatic progenitor formation (Kumar et al., 2014). Effective glucose level control depends on insulin action and the counter-regulation by glucagon signaling, which reciprocally regulates α- and β-cell functions through intra-islet paracrine signaling (Unger and Orci, 2010).

Thus, HGF and EGF seem to contribute to insulin production regulation by pancreatic cells. Interestingly, a weak negative correlation was found between serum levels of HGF and EGF in control subjects of both genders, but this correlation increased in patients with type 2 DM. This study is the first to report a correlation between HGF and EGF release in humans. EGF might also play a significant role in regulating glucagon secretion by counteracting the glucose-induced decrease in plasma glucagon levels, suggesting its potential as a pharmacological agent for type 2 DM patients (Jansen et al., 2006). Similarly, HGF has been proposed as a therapeutic target for insulin resistance, promoting β-cell hyperplasia and hyperinsulinemia (Oliveira et al., 2018). However, our findings indicate that patients with type 2 DM of both genders, particularly women, had elevated serum levels of both HGF and EGF, likely as a compensatory response to normalize glucose metabolism (Table 1). Thus, the administration of exogenous amounts of these growth factors might not offer additional benefits.

Our study revealed a strong positive correlation between serum albumin and HGF levels in women with type 2 DM and no correlation in diabetic men (Figure 3). Conversely, in the control group, men showed a strong correlation (rho coefficient 0.2931), while women had no relationship. The lack of correlation between albumin and EGF remained in diabetic men, but a weak negative correlation was noted in diabetic women. No correlation was found between EGF and albumin in control subjects (Figure 3). Regarding bilirubin, mainly direct bilirubin, a weak and opposite correlation with HGF levels was observed in diabetic patients, with no correlation in control subjects. For EGF, the relationship with direct bilirubin slightly intensified compared to HGF, shifting from no correlation to a weak negative correlation in control subjects and remaining null for diabetic men (Figure 3).

These results suggest that a relationship between markers of liver damage (albumin and bilirubin) and growth factors (HGF and EGF) is apparent primarily in diabetic women. Prior studies on the interactions between nitrogen-related compounds and these growth factors are limited. Recent evidence indicates that red blood cells (RBCs) may regulate serum levels of nitrogen-related metabolites through an extra-hepatic mechanism, with gender-based differences observed between healthy men and women. Moreover, type 2 diabetes mellitus (DM) is associated with increased blood levels of ammonia, citrulline, and MDA, leading to altered nitrogen metabolite management compared to healthy individuals (Contreras-Zentella et al., 2019).

The simultaneous elevation of HGF and EGF levels in patients with metabolic disturbances, along with the observed gender differences, raises questions. The HGF-Met pathway is known for its roles in inhibiting inflammation and fibrotic changes across various cells and tissues. HGF triggers anti-inflammatory mechanisms, such as reducing LPS-induced oxidative stress and inflammation in vascular tissues, through EGFR degradation and angiotensin II signaling inhibition (Kusonoki et al., 2014; Oliveira et al., 2018). In β-cells Met−/− mice, a mild hyperglycemia and loss of acute-phase insulin response to glucose highlight the importance of HGF-Met signaling for normal glucose-dependent insulin secretion and homeostasis (Hartmann et al., 2016). Furthermore, EGF’s role in activating insulin-producing cells through transgenic expression in β-cells suggests its involvement in β-cell activation (Krakowski et al., 1996). EGF also promotes pancreatic ductal cell activation and human β-cell neogenesis (Suarez-Pinzon et al., 2005).

The influence of nitrogen-related compounds on growth factor levels is yet to be fully understood. Our study shows a negligible to weak correlation between serum ammonia and HGF levels in controls, with a weak and opposite trend in diabetic patients (Figure 4). This weak negative relationship between ammonia and EGF was consistent in diabetic men, but ammonia and EGF correlation was negligible in other groups. Additionally, weak correlations between serum nitrites and EGF levels were observed in control women and diabetic men, with no correlation in control men and diabetic women (Figure 4). These findings underscore the impact of gender and pathology on the relationship between growth factor levels and nitrogen metabolism.

The HGF-Met pathway significantly affects glucose and lipid metabolism, with HGF enhancing glucose consumption and lactate production in breast cancer cells (“Warburg effect”). Inhibition of the Met receptor reduces hexokinase expression, suggesting HGF-mediated resistance through glycolysis (Linnemann et al., 2014). HGF, produced by pancreatic β-cells, regulates insulin production to curb hyperglycemia, with its absence leading to reduced insulin levels and mild hyperglycemia in mice (Araújo et al., 2012). HGF levels correlate with body mass index and increase during obesity, potentially serving as an early obesity/insulin resistance signal (Ekberg et al., 1992; Zhou and Hylemon, 2014). In the context of insulin resistance-associated chronic low-grade inflammation, bile acid signaling disruption may lead to impaired lipid metabolism and an increased risk of fatty liver diseases (Balks and Jungermann, 1984). However, our study did not identify a significant role for major blood lipids in growth factor release regulation (Figure 5). In control subjects, correlations between cholesterol, TG, and TGF were weak, except for a moderate negative correlation between TG and HGF in women. Contrarily, a study suggested that HGF levels increase in response to endothelial damage by oxidized LDL-cholesterol, linking higher HGF plasma levels to larger atherosclerotic plaque volumes (Sommer et al., 2024).

Investigations into the relationship between serum HGF levels and type 2 DM severity have shown an inverse correlation with HbA1c levels (Konya et al., 2014). Previous studies reported significantly lower serum HGF levels in type 2 DM patients compared to non-DM subjects, without gender differences (Nakamura, 1991). However, our findings differ, with diabetic men showing no correlation with HGF and diabetic women displaying a weak correlation. A similar pattern was observed for EGF, indicating gender-specific differences.

## 5 Conclusion

Our study highlights the crucial role of insulin and glucagon, pancreatic hormones, as potential regulators of serum glucose and lipid levels, as well as the observed fluctuations in these hormones, primarily *via* their ratio. These factors are intricately connected to changes in two pivotal growth factors, hepatocyte growth factor (HGF) and epidermal growth factor (EGF). Additionally, serum fluctuations in glucose, albumin, bilirubin, ammonia, and nitric oxide (measured as nitrites) may influence the regulation of glucose and lipid levels and are associated with variations in HGF and EGF (see Graphical Abstract). These findings are particularly pronounced in conditions like type 2 diabetes mellitus (DM), characterized by metabolic imbalances and impaired cell proliferation. Dai et al., 2005, Krakowski et al., 1999, Rizwani et al., 2015.

## Data Availability

The datasets presented in this study can be found in online repositories. The names of the repository/repositories and accession number(s) can be found in the article/Supplementary Material.

## References

[B1] Abu Rmilah A. A. Zhou W. Nyberg S. L. (2020). Hormonal contribution to liver regeneration. Mayo Clin. Proc. Innov. Qual. Outcomes, 4(3):315–338. 10.1016/j.mayocpiqo.2020.02.001 32542223 PMC7283948

[B2] Akhtar S. Benter I. F. (2013). The role of epidermal growth factor receptor in diabetes-induced cardiac dysfunction. Bioimpacts 3 (1), 5–9. 10.5681/bi.2013.008 23678464 PMC3648911

[B3] Araújo T. G. Oliveira A. G. Carvalho B. M. Guadagnini D. Protzek A. O. Carvalheira J. B. (2012). Hepatocyte growth factor plays a key role in insulin resistance-associated compensatory mechanisms. Endocrinology 153 (12), 5760–5769. 10.1210/en.2012-1496 23024263

[B4] Balks H. J. Jungermann K. (1984). Regulation of peripheral insulin/glucagon levels by rat liver. Eur. J. Biochem. 141 (3), 645–650. 10.1111/j.1432-1033.1984.tb08240.x 6378634

[B5] Bottaro D. P. Rubin J. S. Faletto D. L. Chan A. M. Kmiecik T. E. Vande Woude G. F. (1991). Identification of the hepatocyte growth factor receptor as the c-met proto-oncogene product. Science 251 (4995), 802–804. 10.1126/science.1846706 1846706

[B6] Breindel J. L. Haskins J. W. Cowell E. P. Zhao M. Nguyen D. X. Stern D. F. (2013). EGF receptor activates MET through MAPK to enhance non-small cell lung carcinoma invasion and brain metastasis. Cancer Res. 73 (16), 5053–5065. 10.1158/0008-5472.CAN-12-3775 23794705 PMC3745527

[B7] Clauset A. Shalizi C. R. Newman M. E. J. (2009). Power-law distributions in empirical data. SIAM Rev. 51 (4), 661–703. 10.1137/070710111

[B8] Contreras-Zentella M. L. Sánchez-Sevilla L. Suárez-Cuenca J. A. Olguín-Martínez M. Alatriste-Contreras M. G. García-García N. (2019). The role of oxidant stress and gender in the erythrocyte arginine metabolism and ammonia management in patients with type 2 diabetes. PLoS One 14 (7), e0219481. 10.1371/journal.pone.0219481 31314811 PMC6636741

[B9] Dai C. Huh C. G. Thorgeirsson S. S. Liu Y. (2005). Beta-cell-specific ablation of the hepatocyte growth factor receptor results in reduced islet size, impaired insulin secretion, and glucose intolerance. Am. J. Pathol. 167 (2), 429–436. 10.1016/s0002-9440(10)62987-2 16049329 PMC1603568

[B10] Ekberg S. Luther M. Nakamura T. Jansson J. O. (1992). Growth hormone promotes early initiation of hepatocyte growth factor gene expression in the liver of hypophysectomized rats after partial hepatectomy. J. Endocrinol. 135 (1), 59–67. 10.1677/joe.0.1350059 1431684

[B11] Fafalios A. Ma J. Tan X. Stoops J. Luo J. Defrances M. C. (2011). A hepatocyte growth factor receptor (Met)-insulin receptor hybrid governs hepatic glucose metabolism. Nat. Med. 17 (12), 1577–1584. 10.1038/nm.2531 22081023 PMC3233634

[B12] Fay M. P. Proschan M. A. (2010). Wilcoxon-Mann-Whitney or t-test? On assumptions for hypothesis tests and multiple interpretations of decision rules. Stat. Surv. 4, 1–39. 10.1214/09-SS051 20414472 PMC2857732

[B13] Fioretto P. Steffes M. W. Sutherland D. E. Goetz F. C. Mauer M. (1998). Reversal of lesions of diabetic nephropathy after pancreas transplantation. New Engl. J. Med. 339 (2), 69–75. 10.1056/NEJM199807093390202 1998 9654536

[B14] Frisch R. N. Curtis K. M. Aenlle K. K. Howard G. A. (2016). Hepatocyte growth factor and alternative splice variants - expression, regulation and implications in osteogenesis and bone health and repair. Expert. Opin. Ther. Targets 20 (9), 1087–1098. 10.1517/14728222.2016.1162293 26941128 PMC5031543

[B15] Hartmann S. Bhola N. E. Grandis J. R. (2016). HGF/Met signaling in head and neck cancer: impact on the tumor microenvironment. Clin. Cancer Res. 22 (16), 4005–4013. 10.1158/1078-0432.CCR-16-0951 27370607 PMC6820346

[B16] Jansen C. Lundquist I. Salehi A. Axelson J. Ohlsson B. (2006). Does epidermal growth factor participate in the regulation of glucose, insulin and glucagon levels? Eur. Surg. Res. 38 (4), 377–384. 10.1159/000094533 16837808

[B17] Jiang J. Au M. Lu K. Eshpeter A. Korbutt G. Fisk G. (2007). Generation of insulin-producing islet-like clusters from human embryonic stem cells. Stem Cells 25(8), 1940–1953. 10.1634/stemcells.2006-0761 17510217

[B18] Joo M. K. Park J. J. Chun H. J. (2016). Recent updates of precision therapy for gastric cancer: towards optimal tailored management. World J. Gastroenterol. 22 (19), 4638–4650. 10.3748/wjg.v22.i19.4638 27217696 PMC4870071

[B19] Kawaguchi M. Kataoka H. (2014). Mechanisms of hepatocyte growth factor activation in cancer tissues. Cancers (Basel) 6 (14), 1890–1904. 10.3390/cancers6041890 25268161 PMC4276949

[B20] Konya H. Miuchi M. Satani K. Matsutani S. Tsunoda T. Yano Y. (2014). Hepatocyte growth factor, a biomarker of macroangiopathy in diabetes mellitus. World J. Diabetes 5 (5), 678–688. 10.4239/wjd.v5.i5.678 25317245 PMC4138591

[B21] Krakowski M. L. Kritzik M. R. Jones E. M. Krahl T. Lee J. Arnush M. (1999). Transgenic expression of epidermal growth factor and keratinocyte growth factor in beta-cells results in substantial morphological changes. J. Endocrinol. 162 (2), 167–175. 10.1677/joe.0.1620167 10425454

[B22] Kroy D. C. Schumacher F. Ramadori P. Hatting M. Bergheim I. Gassler N. (2014). Hepatocyte specific deletion of c-Met leads to the development of severe non-alcoholic steatohepatitis in mice. J. Hepatol. 61 (4), 883–890. 10.1016/j.jhep.2014.05.019 24845607

[B23] Kumar S. S. Alarfaj A. A. Munusamy M. A. Singh A. J. Peng I. C. Priya S. P. (2014). Recent developments in β-cell differentiation of pluripotent stem cells induced by small and large molecules. Int. J. Mol. Sci. 15 (12), 23418–23447. 10.3390/ijms151223418 25526563 PMC4284775

[B24] Kusunoki H. Taniyama Y. Otsu R. Rakugi H. Morishita R. (2014). Anti-inflammatory effects of hepatocyte growth factor on the vicious cycle of macrophages and adipocytes. Hypertens. Res. 37 (6), 500–506. 10.1038/hr.2014.41 24621470

[B25] Linnemann A. K. Baan M. Davis D. B. (2014). Pancreatic β-cell proliferation in obesity. Adv. Nutr. 5 (3), 278–288. 10.3945/an.113.005488 24829474 PMC4013180

[B26] Mann H. B. Whitney D. R. (1947). On a test of whether one of two random variables is stochastically larger than the other. Ann. Math. Stat. 18, 50–60. 10.1214/aoms/1177730491

[B27] Marquardt J. U. Seo D. Gómez-Quiroz L. E. Uchida K. Gillen M. C. Kitade M. (2012). Loss of c-Met accelerates development of liver fibrosis in response to CCl(4) exposure through deregulation of multiple molecular pathways. Biochim. Biophys. Acta. 1822 (6), 942–951. 10.1016/j.bbadis.2012.02.012 22386877 PMC3338880

[B28] Mellado-Gil J. Rosa T. C. Demirci C. Gonzalez-Pertusa J. A. Velazquez-Garcia S. Valle S. (2011). Disruption of hepatocyte growth factor/c-Met signaling enhances pancreatic beta-cell death and accelerates the onset of diabetes. Diabetes 60 (2), 525–536. 10.2337/db09-1305 20980460 PMC3028352

[B29] Nakamura S. Morishita R. Moriguchi A. Yo Y. Nakamura Y. Hayashi S. (1998). Hepatocyte growth factor as a potential index of complication in diabetes mellitus. J. Hypertens. 16 (12 Pt 2), 2019–2026. 10.1097/00004872-199816121-00025 9886892

[B30] Nakamura T. (1991). Structure and function of hepatocyte growth factor. Prog. Growth Factor Res. 3 (1), 67–85. 10.1016/0955-2235(91)90014-u 1838014

[B31] Naldini L. Vigna E. Narsimhan R. P. Gaudino G. Zarnegar R. Michalopoulos G. K. (1991). Hepatocyte growth factor (HGF) stimulates the tyrosine kinase activity of the receptor encoded by the proto-oncogene c-MET. Oncogene 6 (4), 501–504. 1827664

[B32] Okunishi K. Dohi M. Nakagome K. Tanaka R. Mizuno S. Matsumoto K. (2005). A novel role of hepatocyte growth factor as an immune regulator through suppressing dendritic cell function. J. Immunol. 175 (7), 4745–4753. 10.4049/jimmunol.175.7.4745 16177122

[B33] Oliveira A. G. Araújo T. G. Carvalho B. M. Rocha G. Z. Santos A. Saad M. J. A. (2018). The role of hepatocyte growth factor (HGF) in insulin resistance and diabetes. Front. Endocrinol. (Lausanne) 9, 503. 10.3389/fendo.2018.00503 2018, Aug30 30214428 PMC6125308

[B34] Otonkoski T. Cirulli V. Beattie M. Mally M. I. Soto G. Rubin J. S. (1996). A role for hepatocyte growth factor/scatter factor in fetal mesenchyme-induced pancreatic beta-cell growth. Endocrinology 137 (7), 3131–3139. 10.1210/endo.137.7.8770939 8770939

[B35] Ramírez-Zamora S. Méndez-Rodríguez M. L. Olguín-Martínez M. Sánchez-Sevilla L. Quintana-Quintana M. García-García N. (2013). Increased erythrocytes by-products of arginine catabolism are associated with hyperglycemia and could be involved in the pathogenesis of type 2 diabetes mellitus. PLoS One 8 (6), e66823. 10.1371/journal.pone.0066823 23826148 PMC3691261

[B36] Rizwani W. Allen A. E. Trevino J. G. (2015). Hepatocyte growth factor from a clinical perspective: a pancreatic cancer challenge. Cancers (Basel) 7 (3), 1785–1805. 10.3390/cancers7030861 26404380 PMC4586794

[B37] Siddiqui S. Fang M. Ni B. Lu D. Martin B. Maudsley S. (2012). Central role of the EGF receptor in neurometabolic aging. Int. J. Endocrinol. 2012, 739428. 10.1155/2012/739428 22754566 PMC3382947

[B38] Sommer P. Schreinlechner M. Noflatscher M. Engl C. Lener D. Theurl M. (2024). Hepatocyte growth factor as indicator for subclinical atherosclerosis. Vasa 2024. 10.1024/0301-1526/a001111 Jan 11 38205733

[B39] Song I. Patel O. Himpe E. Muller C. J. Bouwens L. (2015). Beta cell mass restoration in alloxan-Diabetic mice treated with EGF and gastrin. PLoS One 10 (10), e0140148. 10.1371/journal.pone.0140148 26452142 PMC4599944

[B40] Suarez-Pinzon W. L. Lakey J. R. Brand S. J. Rabinovitch A. (2005). Combination therapy with epidermal growth factor and gastrin induces neogenesis of human islet {beta}-cells from pancreatic duct cells and an increase in functional {beta}-cell mass. J. Clin. Endocrinol. Metab. 90, 3401–3409. 10.1210/jc.2004-0761 15769977

[B41] Suzuki M. Shiraha H. Fujikawa T. Takaoka N. Ueda N. Nakanishi Y. (2005). Des-gamma-carboxy prothrombin is a potential autologous growth factor for hepatocellular carcinoma. J. Biol. Chem. 280 (8), 6409–6415. 10.1074/jbc.M406714200 15582995

[B42] Tebar F. Soley M. Ramírez I. (1996). The antilipolytic effects of insulin and epidermal growth factor in rat adipocytes are mediated by different mechanisms. Endocrinology 137 (10), 4181–4188. 10.1210/endo.137.10.8828475 8828475

[B43] Unger R. H. Orci L. (2010). Paracrinology of islets and the paracrinopathy of diabetes. Proc. Natl. Acad. Sci. U. S. A. 107, 16009–16012. 10.1073/pnas.1006639107 20798346 PMC2941311

[B44] Wasserstein R. L. Lazar N. A. (2016). The ASA statement on *p*-values: context, process, and purpose. Am. Stat. 70 (2), 129–133. 10.1080/00031305.2016.1154108

[B45] Wild S. Roglic G. Green A. Sicree R. King H. (2004). Global prevalence of diabetes: estimates for the year 2000 and projections for 2030. Diabetes Care 27 (5), 1047–1053. 10.2337/diacare.27.5.1047 15111519

[B46] Yamada T. Hisanaga M. Nakajima Y. Mizuno S. Matsumoto K. Nakamura T. (2000). Enhanced expression of hepatocyte growth factor by pulmonary ischemia-reperfusion injury in the rat. Am. J. Respir. Crit. Care Med. 162 (2 Pt 1), 707–715. 10.1164/ajrccm.162.2.9908064 10934110

[B47] Zhang D. Jiang W. Liu M. Sui X. Yin X. Chen S. (2009). Highly efficient differentiation of human ES cells and iPS cells into mature pancreatic insulin-producing cells. Cell. Res. 19 (4), 429–438. 10.1038/cr.2009.28 19255591

[B48] Zhang Y. Jain R. K. Zhu M. (2015). Recent progress and advances in HGF/MET-targeted therapeutic agents for cancer treatment. Biomedicines 3 (1), 149–181. 10.3390/biomedicines3010149 28536405 PMC5344234

[B49] Zhou H. Hylemon P. B. (2014). Bile acids are nutrient signaling hormones. Steroids 86, 62–68. 10.1016/j.steroids.2014.04.016 24819989 PMC4073476

