# Supplementary material for: Gender effect of glucose, insulin/glucagon ratio, lipids, and nitrogen-metabolites on serum HGF and EGF levels in patients with diabetes type 2
Source: Front Mol Biosci. 2024 Apr 9;11:1362305. doi: 10.3389/fmolb.2024.1362305 (PMC11035728; doi:10.3389/fmolb.2024.1362305)
Supplement: Supplementary file 2 [file Table2.DOCX]

**S1. Best fits distributions**

| **Variable** | **Bestfit** | **KS-test** | | **MLEs** | | | |
| --- | --- | --- | --- | --- | --- | --- | --- |
|  | **distribution** | **D-statistic** | **p-value** |  | | | |
| Albumin**_**Cirrhosis_  Ma | beta | 0.099364978052685293 | 0.82454008364820996 | 1.8031487112697588 | 1.1843385374214956 | 2.2106585310703202 | 1.7957929081723665 |
| Albumin_  Control_  Ma | beta | 0.11081172642991088 | 0.6974382447142693 | 2.1850636619363266 | 1.6204092205820473 | 3.0984829529348881 | 2.951312056042763 |
| Albumin_  Control_  Fe | Exponentialweibull | 0.078461911940195805 | 0.96630120158943633 | 0.13537683749916418 | 8.3917110466614897 | 3.3934061829190991 | 1.4662660504658758 |
| Albumin_  DM_Ma | Exponentialweibull | 0.070075328707196349 | 0.98941356703315642 | 0.15934823513663404 | 10.507021675418418 | 2.7125420443796031 | 2.1349579883157377 |
| Albumin_  DM_Fe | weibull_min | 0.083355800432593274 | 0.94385357421661231 | 5.9370985970134402 | 1.2461035354228009 | 3.0573236181530126 |  |
| Direct  Bilirubin__Control_  Ma | logistic | 0.11582627544722675 | 0.63467504803970787 | 0.18120946598052978 | 0.034022360857934891 |  |  |
| Direct  Bilirubin_  Control_  Fe | weibull_max | 0.12051699252531078 | 0.57894753315034464 | 7.9941594196846335 | 0.81688783204607218 | 0.63475187182561399 |  |
| Direct  Bilirubin_  DM_  Ma | logistic | 0.075299791472251659 | 0.97714907881856961 | 0.23407261567711835 | 0.029116504200762415 |  |  |
| Direct  Bilirubin_  DM_  Fe | weibull_max | 0.08284452063611894 | 0.94652809703308882 | 13.04433759115318 | 0.47465167300163935 | 0.37984724332957664 |  |
| Total Bilirubin_  Control_  Ma | logistic | 0.090024145341210615 | 0.90206970403325448 | 0.62828013425320406 | 0.087126883631661461 |  |  |
| Total Bilirubin_  Control_  Fe | Exponentialpowerlaw | 0.12953961247747781 | 0.48028269694324011 | 0.95802748030707807 | 0.24999999999999997 | 0.37363187519157315 |  |
| Total Bilirubin_  DM_  Ma | weibull_max | 0.067886205333432315 | 0.99275941217769004 | 4.039276323661003 | 0.48592758682283915 | 0.1942684500193344 |  |
| Total Bilirubin_  DM_  Fe | beta | 0.082577362828698542 | 0.94789495958680636 | 1.3948701750257415 | 2.0024134112566285 | 0.067972755114967781 | 0.21603664899321243 |
| Choleste-  rol_  Control_  Ma | logistic | 0.090658972438579677 | 0.89745109483425023 | 138.24931022985305 | 7.8713311254100189 |  |  |
| Choleste-rol_  Control_  Fe | logistic | 0.073120665575718433 | 0.98306621579965792 | 148.64394973549631 | 9.1103365756719015 |  |  |
| Choleste-rol_  DM_Ma | logistic | 0.09502613675456556 | 0.86295847141137638 | 175.27362581028748 | 13.752729558193851 |  |  |
| Choleste-rol_  DM_Ma | beta | 0.067709555416287093 | 0.99298997054454607 | 1.9485887457112971 | 1.5685151658306229 | 143.59028959016109 | 74.925014378935231 |
| EGF_  Control_  Ma | weibull_min | 0.072395634732287772 | 0.98477172103830579 | 3.2421673314055131 | 0.23917586083365272 | 1.2776801227628933 |  |
| EGF_  Control_  Fe | Exponentialweibull | 0.11528875851957943 | 0.64124788606844563 | 0.18960058890951553 | 6.7888185072429605 | 0.68983918281693046 | 0.85149606126249711 |
| EGF_DM_  Ma | Exponentialpowerlaw | 0.085484411038681513 | 0.93189445932660031 | 1.6715223107171673 | 0.73138117024700566 | 1.5787553465450688 |  |
| EGF_DM_  Fe | beta | 0.11165689467392548 | 0.68663646298543801 | 0.74883728748688694 | 0.75814789857531772 | 1.3955383756337032 | 1.4044616243662968 |
| Glucagon_Control_  Ma | powerlaw | 0.095790144335270916 | 0.85647014111352093 | 1.025614890880826 | 21.181208947685967 | 28.418791256674865 |  |
| Glucagon_Control_Fe | powerlaw | 0.091799911170821424 | 0.88888879743040106 | 0.92812370222057528 | 21.399999999999995 | 33.612579033675303 |  |
| Glucagon_DM_Ma | beta | 0.056360170498439943 | 0.99957319968140468 | 1.7307038329435991 | 1.5049726522980085 | 20.3651104695284 | 18.325144159115883 |
| Glucagon_DM_Fe | logistic | 0.064667334575504193 | 0.99616008383844756 | 31.370890440766573 | 2.4866868760682745 |  |  |
| Glucose**_**  Control_  Ma | beta | 0.066488671353012174 | 0.99443661380517578 | 1.3080556507522143 | 2.228276396322534 | 117.46346521215393 | 88.21505301537151 |
| Glucose_  Control_  Fe | lognormal | 0.056184065779072645 | 0.99959718321177315 | 0.2569844469211498 | 76.460632754515515 | 65.363728990195526 |  |
| Glucose_  DM_Ma | powerlaw | 0.072821242806068831 | 0.98378594431699085 | 1.0503315544364957 | 110.83600096980945 | 117.16399904116679 |  |
| Glucose_  DM_Fe | logistic | 0.12537822785665176 | 0.5243675007596269 | 180.32412086684892 | 18.771454092377226 |  |  |
| HGF_  Control_  Ma | beta | 0.12303678748285601 | 0.55024628171880963 | 1.5182296327863527 | 1.4435481878703973 | 4.0464482433083067 | 8.9859617844318826 |
| HGF_  Control_  Fe | beta | 0.05296493259449131 | 0.99987432496140327 | 2.0103565100863774 | 2.3808393065242157 | 3.435726235924669 | 12.597039472126415 |
| HGF_DM_Ma | exponentialpowerlaw | 0.075983740095968755 | 0.97503601898609904 | 1.495943 | 7184090345 | 5.1815166560946029 | 16.993870494759882 |
| HGF_DM_  Fe | Exponentialweibull | 0.076551709830476655 | 0.97318496206286975 | 33.692942783177784 | 4.3522104006946503 | -85.079515095213026 | 76.716066555578976 |
| Insulin_  Control_  Ma | gilbrat | 0.1052792981056267 | 0.77027253797919037 | 0.80466716677299766 | 3.9642703426780441 |  |  |
| Insulin_  Control_  Fe | Exponentialweibull | 0.10652752873175297 | 0.75352676074713054 | 0.54911926943232015 | 1.6081461296582793 | 0.89999999999999991 | 11.171508617797514 |
| Insulin_  DM_  Ma | exponentalpowerlaw | 0.10763362428626033 | 0.73883713800173378 | 0.86535792266914457 | 0.89999999999999991 | 15.99069637373173 |  |
| Insulin_  DM_  Fe | exponentialpowerlaw | 0.11440802219086182 | 0.65209913362864591 | 0.90456620109096519 | 0.59999999999999987 | 15.937329905210767 |  |
| Insulin/  Glucagon_Control_  Ma | lognormal | 0.10588160592554596 | 0.7621701688072815 | 0.81154579682614325 | 0.010121443840569431 | 0.13364477214735565 |  |
| Insulin/  Glucagon_Control_Fe | exponentialpowerlaw | 0.10201138592425579 | 0.79944259484051516 | 0.8999599804968329 | 0.025423728813559317 | 0.35267560160959843 |  |
| Insulin/  Glucagon_DM_Ma | weibull_min | 0.10357616842517425 | 0.79340102808282875 | 0.9416831195017914 | 0.042857142857142851 | 0.31140524645402012 |  |
| Insulin/  Glucagon_DM_Fe | lognormal | 0.088853372929518282 | 0.91030679587658825 | 0.65175303354730052 | -0.062098852041595029 | 0.3215759516556993 |  |
| Triglyce-rides_  Control_  Ma | beta | 0.066488671353012174 | 0.99443661380517578 | 1.3080556507522143 | 2.228276396322534 | 117.46346521215393 | 88.21505301537151 |
| Triglyce-rides_  Control_  Fe | lognormal | 0.056184065779072645 | 0.99959718321177315 | 0.2569844469211498 | 76.460632754515515 | 65.363728990195526 |  |
| Triglyce-rides_DM_Ma | powerlaw | 0.072821242806068831 | 0.98378594431699085 | 1.0503315544364957 | 110.83600096980945 | 117.16399904116679 |  |
| Triglyce-rides_  DM_Fe | logistic | 0.12537822785665176 | 0.5243675007596269 | 180.32412086684892 | 18.771454092377226 |  |  |
| Nitrites_  Control_  Ma | exponentialpowerlaw | 0.06624267838030784 | 0.99469844204570934 | 1.4599326649126925 | 4.6603337121492245 | 22.702231623755175 |  |
| Nitrites_  Control_  Fe | lognormal | 0.062258553678150766 | 0.99777073865062949 | 0.49811572246119007 | 2.329513264149579 | 10.634233107458794 |  |
| Nitrites_  DM_Ma | weibull_min | 0.068795660196611597 | 0.99148210365777023 | 1.448737044678797 | 9.3528472365662552 | 46.427537223073372 |  |
| Nitrites_  DM_  Fe | beta | 0.098994915391336202 | 0.82795717406520719 | 0.76741471044279597 | 0.6879665768623151 | 20.127316170753978 | 63.272683829246034 |
| HBAC_  Control_  Ma | beta | 0.073644255310878592 | 0.98175465408459084 | 1.8045028274430315 | 1.7820899217365747 | 16.158085213537262 | 23.633108718379699 |
| HBAC_  Control_  Fe | Powerlaw | 0.086465614739284158 | 0.92593873890288148 | 1.0792485599426098 | 16.965381553990262 | 15.034618533350297 |  |
| HBAC_  DM_  Ma | beta | 0.066730123904769137 | 0.99417021910507453 | 1.7937508978429872 | 1.72385242270454 | 39.949391327837425 | 58.865814664549305 |
| HBAC_  DM  Fe | Powerlaw | 0.086466546922252863 | 0.9259329494108014 | 1.079250760160317 | 50.895864714891673 | 45.104135331675622 |  |
| HOMA_  Control_  Ma | 'weibull_max | 0.097530726411460933 | 0.84123264377349249 | 2.7938987643066744 | 1.949923878655456 | 0.56341451864385939 |  |
| HOMA_  Control_  Fe | 'beta' | 0.098560479015289471 | 0.83193750785176346 | 0.80008367423413163 | 0.74105063253226999 | 1.2675437617766199 | 0.77245623822338039 |
| HOMA_  DM_Ma | Expweibull' | 0.077885438825262554 | 0.96848702246662099 | 0.18866169329160579 | 8.4334236450083768 | 1.9526028138362346 | 1.3827505011557455 |
| HOMA_  DM_Fe | 'beta' | 0.1005145056786544 | 0.81377605259260044 | 1.0298205677681851 | 0.69066797131294777 | 1.78502011614 | 1.7849798838592852 |
| Amm_  Control_Ma, | Logistic | 0.0713760183432732 | 0.977993807309539 | 5.07684749947388 | 1.13781617019794 |  |  |
| Amm_  Control_Fe, | Beta | 0.121702454018753 | 0.553133205838336 | 1.02512103632583 | 0.674684149910576 | 1.14351747761873 | 5.45648252238127 |
| Amm_  DM_Ma | Weibull minumum | 0.119979353358421 | 0.57114447321451 | 0.910641986340272 | 3.5 | 6.32200939027367 |  |
| Amm_  DM_Fe | Logistic | 0.152254549524531 | 0.282214883589322 | 9.51419574507591 | 1.93046891920449 |  |  |

Ma: male.

Fe: female.

DM: Diabetic patients type 2

HBAC: Hb A_IC_

Amm: Ammonium
