# Supplementary material for: Gender effect of glucose, insulin/glucagon ratio, lipids, and nitrogen-metabolites on serum HGF and EGF levels in patients with diabetes type 2
Source: Front Mol Biosci. 2024 Apr 9;11:1362305. doi: 10.3389/fmolb.2024.1362305 (PMC11035728; doi:10.3389/fmolb.2024.1362305)
Supplement: Supplementary file 4 [file Table3.DOCX]

**Supplement 3**,

| **Determination** | **Statistic** | **p-value** | **Distribution Between Gender** |
| --- | --- | --- | --- |
| **Control**  Cholesterol | 527.5 | 0.008836 | Since the p-value <0.05 we reject the null hypothesis in favor of the alternative. Therefore, the distributions are **statistically different**. |
| **Control**  Glucagon | 667.0 | 0.2022416087739164 | Since the p-value >0.05 we do not reject the null hypothesis in favor of the alternative. Therefore, the distributions are **statistically the same** |
| **DM**  Insulin | 806.5 | 0.9539540320197264) | Since the p-value >0.05 we do not reject the null hypothesis in favor of the alternative.  Therefore, the distributions are **statistically the** **same**.  We highlight the fact that the p-  value is very high; consequently, we do not reject the null hypothesis at any confidence interval, not even 5%. |

**S3.** Mann-Whitney U test. With a p-value <0.05 we reject the null hypothesis and the distributions are statistically different.
